# Genomic approaches for monitoring transmission dynamics of malaria: A case for malaria molecular surveillance in Sub–Saharan Africa

**DOI:** 10.3389/fepid.2022.939291

**Published:** 2022-10-28

**Authors:** Benedicta A. Mensah, Nukunu E. Akyea-Bobi, Anita Ghansah

**Affiliations:** ^1^Department of Epidemiology, Noguchi Memorial Institute for Medical Research, University of Ghana, Accra, Ghana; ^2^Department of Parasitology, Noguchi Memorial Institute for Medical Research, University of Ghana, Accra, Ghana

**Keywords:** malaria, transmission, genomics, techniques, surveillance

## Abstract

Transmission dynamics is an important indicator for malaria control and elimination. As we move closer to eliminating malaria in Sub-Saharan Africa (sSA), transmission indices with higher resolution (genomic approaches) will complement our current measurements of transmission. Most of the present programmatic knowledge of malaria transmission patterns are derived from assessments of epidemiologic and clinical data, such as case counts, parasitological estimates of parasite prevalence, and Entomological Inoculation Rates (EIR). However, to eliminate malaria from endemic areas, we need to track changes in the parasite population and how they will impact transmission. This is made possible through the evolving field of genomics and genetics, as well as the development of tools for more in-depth studies on the diversity of parasites and the complexity of infections, among other topics. If malaria elimination is to be achieved globally, country-specific elimination activities should be supported by parasite genomic data from regularly collected blood samples for diagnosis, surveillance and possibly from other programmatic interventions. This presents a unique opportunity to track the spread of malaria parasites and shed additional light on intervention efficacy. In this review, various genetic techniques are highlighted along with their significance for an enhanced understanding of transmission patterns in distinct topological settings throughout Sub-Saharan Africa. The importance of these methods and their limitations in malaria surveillance to guide control and elimination strategies, are explored.

## Introduction

Despite increased attempts to manage the disease, malaria still has a high mortality and morbidity rate throughout Africa (1). The most recent figures show that the number of malaria cases and deaths increased by 5 and 12%, respectively, in 2020 from 2019 (1). Due to varying transmission intensities among communities, the malaria burden is highly variable throughout Africa (2). Malaria transmission is a dynamic process that is impacted by a variety of interconnected factors, such as interventional pressures, uncontrollable natural environmental conditions, and human-caused disruptions of the environment (3). In addition, a single symptomatic human with a high *de novo* mutation rate (4) has the potential to continuously generate genetic variation across the millions of symptomatic and asymptomatic cases recorded annually. These mutations make it difficult to ascertain the genetic make-up of the transmitted parasites in subsequent generations. Furthermore, the infections may also be polyclonal, thus making the parasite highly adaptable and challenging to analyze.

For decades, epidemiological and clinical data analysis, such as prevalence rates and case counts (5, 6), the entomological index known as the Entomological Inoculation Rate (EIR) (7), or parasitological parameters have been used to determine the level of malaria transmission (6). To eradicate malaria, however, surveillance systems must be improved (8) and reliable data for surveillance will come from a more precise assessment of transmission patterns (9, 10). New tools will also be required to improve diagnosis, characterize, and understand the dynamics of the parasite reservoir as it evades host immunity and responds to pressures exerted by interventions (10). As the field is evolving, the tools for measuring transmission intensity and dynamics are also improving with higher resolution. These have been made possible through the growing field of genomic epidemiology and tool development, which are both fueled by advances in the computational analysis of the *Plasmodium* genome (11). In addition to defining prevalence and incidence with higher resolution than the traditional approaches, genomic technologies for tracking parasite dynamics and informing transmission levels also offer novel and potent ways to quantify genetic indices.

Genetic epidemiology is useful for monitoring; the origin and spread of infections, the effectiveness of interventions like antimalarial drugs and vaccines, and the prevalence of the asymptomatic reservoir of infection which drives transmission. Genomic epidemiology has been used to identify relationships between the genetic diversity of the malaria parasite, dynamic changes in transmission intensity, and the effectiveness of malaria control programs (12–15). In Senegal, a validated molecular barcode with 24 Single Nucleotide Polymorphisms (SNPs) was utilized to track distinct parasite types to monitor changes in transmission intensity (16). More specifically, genomic tools can provide information about the parasite population structure at the local level. Population structure can show whether particular genotypes predominate hotspots from the local transmission of specific strains or whether the landscape of malaria transmission is more genetically diverse. This will have significant potential for outcrossing resulting from sustained transmission or importation of multiple genotypes (16–18). Genomic epidemiology can reveal parasite genetic features underlying local and regional patterns of transmission such as Complexity of Infection (COI), diversity (19), parasite population migration and evolution which cannot be deciphered using conventional measures. Furthermore, genomic data can provide information for characterizing connectivity and transmission of different parasite strains (12, 18). Studies on the declining local transmission in the Thailand–Myanmar border region found that the strongest genetic signal associated with transmission decline was loss of parasite genetic diversity within hosts, as measured by a decline in COI (20). Thus, suggesting that this measure could be a proxy for local transmission intensity (21).

When combined with epidemiological and Geographic Information Systems (GIS) data, genetic information may be utilized to identify transmission hotspots and customize interventions. As a result, comprehensive data on the dynamics of the parasite population in a particular area will become available, allowing for more precisely targeted interventions (17, 22). Other molecular epidemiological parameters used to define the infection dynamics of *P. falciparum* include the rate at which different genotypes are acquired over time (molecular force of infection, molFOI). In Papua New Guinea, molFOI was used to describe recrudescent *Plasmodium vivax* infection as opposed to vector-acquired infection and the length of infection (23, 24). If these new tools are incorporated programmatically, surveillance of *Plasmodium* parasites can give important information about malaria transmission and can be used to inform decision-making processes within National Malaria Control Programs (NMCPs) (25).

Parasite genotyping to investigate transmission dynamics during malaria surveillance can be done in several ways (Table 1). For example, high-resolution melting assays and polymerase chain reaction for SNP genotyping (PCR). Even though genotyping of vaccine candidate antigens, such as Circumsporozoite Protein (*csp*), Apical Membrane Antigen 1 (*ama1*), and Merozoite Surface Proteins 1 and 2 (*msp*1&2), have been shown to have the potential to pinpoint the major routes and reservoirs of infection, immune selection limits the viability of this method (26–29). These genes have also been used to assess the causes and dynamics of malaria outbreaks, to monitor imported malaria and its role in local transmission, and to predict the frequency of *P. vivax* relapses (25). However, there are still restrictions on how these genotyping approaches may be used to distinguish between reinfection and recrudescence in order to correctly define the *Plasmodium* population and to detect low parasitaemia in an asymptomatic human host (26). Some of these methods still have limitations when it comes to their capacity to provide high-throughput data for whole-genome information. For instance, novel structural mutations important to downstream applications cannot be found by PCR genotyping (30).

**Table 1:** Genomic approaches for monitoring transmission dynamics and their advantages.

**Approaches**	**Measures of transmission dynamics**	**Advantages**	**Disadvantages**
Antigenic variation SNPS	1. Complexity of Infection (COI). 2. Recrudescence from reinfection. 3. Determining genetic diversity.	1. The availability of PCR and DNA electrophoresis tools has made antigen-coding gene genotyping one of the most widely utilized methods.	1. Absence of standardization of scoring and reporting. 2. Labor-intensive and dependent on the sensitivity of PCR. 3. Genes are subject to immunological selection, which can bias the frequency of certain allelotypes.
SNPs	1. Complexity of Infection (COI). 2. Differentiating recrudescence from reinfection. 3. Determining genetic diversity. 4. Determining within-host diversity.	1. SNP-based genotyping approaches have an advantage over microsatellites in that SNP data are inexpensive, easy to obtain from commonly used dried blood spot (DBS) samples, and suitable for field samples across a range of transmission settings. Thus, SNP data remain the most frequently used approach for genotyping studies.	1. It limits analyses to what is known, requires the design of primers, data is primarily interpreted per SNP. 2. Difficulty in detecting relevant structural mutations. 3. Requires advanced PCR and Array technology.
Microsatellites	1. Complexity of Infection (COI). 2. Differentiating recrudescence from reinfection. 3. Determining genetic diversity. 4. Determining within-host diversity.	1. This method outperformed surface antigen-based genetic typing due to its neutral evolving nature, simplicity of multiplexing with 10 or more markers, and its abundance throughout the genome.	1. No method exists for resolving parasite haplotypes in mixed infections and standardization across laboratories is complicated due to the variety of methods used in genotyping. 2. Introduction of artifacts during PCR amplification, making population structure studies painstaking. 3. The scarcity of markers, the high mutation rate, and the difficulty of appropriately scoring alleles reduce the resolution of microsatellite markers used to identify related parasites.
Amplicon targeted deep sequencing	1. Complexity of Infection (COI). 2. Differentiating recrudescence from reinfection. 3. Determining genetic diversity. 4. Determining within-host diversity.	1. Multiple loci of genome-wide distribution can be sequenced, which is useful for population genetic studies because these loci are unrelated. 2. It can be multiplexed, is cost-effective, requires less time, and, most significantly, offers a high degree of precision and specificity.	1. Non-availability of general standards to correct sequencing errors that may arise. 2. Methodological accuracy issues during the experimental procedure, guaranteeing equimolar concentrations of amplicons to minimize over-representation of certain clones per sample, and limitations of detection for minority clones based on known DNA controls.
Whole genome sequencing	1. Complexity of Infection (COI). 2. Differentiating recrudescence from reinfection. 3. Determining genetic diversity. 4. Determining within-host diversity.	1. Genome-wide diversity inferences are less subject to target bias and hence more accurately reflect broad patterns of P. falciparum genetic variation than individual locus analyses, which may be sensitive to strong natural selection.	1. Very expensive. 2. Technical constraints connected with large-scale data storage and processing.

Microsatellite genotyping, which entails amplification and sequencing, or gel-based characterization of tandem repeats and outputting length polymorphism is another widely used approach. Microsatellites were used to identify the intercontinental spread of *P. falciparum* antimalarial drug resistance alleles from Asia to Africa (31) and more recently, across the eastern greater Mekong sub-region (32, 33). Whole genome sequencing (WGS) employing next-generation sequencing (NGS) technology was developed in response to the need for improved resolution and more coverage of genomes following the sequencing of the *Plasmodium* genome. WGS is crucial for discovering novel genetic variations relevant for surveillance and validating improvements in genomic methodology. Higher sequence reads per instrument run at lower costs are produced by NGS technologies (27, 30). Despite the significant decrease in sequencing costs over the past two decades, large-scale WGS of parasites for surveillance purposes is not practical, particularly in Sub-Saharan Africa (sSA). The research community has been actively evaluating the potential role of targeted sequencing and genotyping approaches to recover the most informative genomic regions for malaria genomic epidemiology (25, 34, 35).

The choice of genotyping technique varies for different endemic settings depending on the priority and cost involved in these approaches. The selection of an appropriate approach for certain endemic settings continues to be a challenge, especially for nations in sSA with limited access to new technologies. For countrywide molecular surveillance in sSA to be successful, a combination of techniques and approaches derived from the technological advancement and adaptation to the evolving epidemiology of malaria will be required. This paper reviews current approaches and tools for determining *P. falciparum* transmission dynamics and offers suggestions for how the various approaches could be applied in resource-limited contexts (29). The importance of these techniques and their limitations in incorporating them into malaria surveillance to guide control and elimination strategies, are discussed.

The research articles were chosen by a search conducted in PubMed and Google Scholar using relevant key terms (malaria, transmission dynamics and the genomic approaches). A review of several papers that met our inclusion criteria uncovered other papers containing relevant information. In all, 94 papers on tool development, assessment of the tools and practical uses of the genomic approaches were included in this review.

## Genomic approaches and tools for measuring malaria transmission dynamics

### Genotyping of antigen-coding gene

*Plasmodium* parasites express a large number of different antigens on the surface of their cells. These variations are critical for virulence and host immune evasion (29, 36, 37). They are suitable markers for the identification of genetically distinct *P. falciparum* parasite sub-populations (29). Earlier studies in genotyping *Plasmodium* infection used length polymorphisms in antigen-coding genes like *msp1, msp2*, and glutamate-rich protein (*glurp*) to determine COI (a measure of the effectiveness of intervention programmes), genetic diversity and to differentiate between recrudescence and reinfection during drug efficacy trials (38–40). Genotyping of antigen-coding genes has become one of the most extensively used approaches due to the availability of PCR and DNA electrophoresis equipment (35). For example, Huang et al. in 2018 (41) and Papa Mze et al. in 2022 (42) demonstrated, by *msp1&2* genotyping, a reduction in COI and genetic diversity in the Grande Comore Island, as a result of a drastic drop in transmission caused by past interventions.

Although this approach has shown utility, it has limitations such as the absence of standardization of scoring and reporting formats that enable the comparison of results among laboratories in various endemic sites. In addition, the *msp/glurp* genotyping process is labor-intensive and is dependent on the sensitivity of PCR, which may fail to amplify low-abundance variants or produce artifacts (43). Moreover, sensitivity is low when agarose gels are used, and interpretation is subjective, particularly in high-transmission areas of Africa, where polyclonal infections result in many bands. Additionally, the *msp/glurp* genes are subject to immunological selection, which can bias the frequency of certain allelotypes (35, 44). This may affect the precision with which population structure and transmission patterns derived from these loci are estimated (35).

It is difficult to interpret population structure using data produced from these loci because it is unclear whether the observed patterns reflect population history or natural selection. Capillary gel separation has been demonstrated to be three times faster, with superior resolution (2.4 x), and separation efficiency (5.4 x) than a typical gel DNA separation (45). Gupta and colleagues (46) demonstrated that capillary electrophoresis provides more accurate outcomes for anti-malarial treatment trials in determining whether recurrent parasitaemia following therapy indicates recrudescence (treatment failure) or a new infection. Although this technique is an update from gel electrophoresis to discriminate *P. falciparum* infections, it requires an expensive equipment and is not as readily available as gel electrophoresis (46). Current WHO recommendations suggest that all studies in sSA should employ *msp1* and *msp2* along with a panel of two to three informative microsatellite markers (such as *Poly-*α, *Pfpk2* and *TA1*) and use the match-counting method for analysis (47).

### Microsatellite genotyping approach

Microsatellites are highly polymorphic tandem repeats of one to six base pairs (bp). They occur often in the *P. falciparum* genome, mostly as [TA]n, [T]n, and [TAA]n repeats (35, 48). Microsatellite markers are extremely abundant in *P. falciparum*, appearing around every 2–3 kb throughout the genome (48, 49). They are considered “selectively neutral” loci, thus, powerful markers for population genetic studies. Microsatellites can be utilized to fingerprint parasites and resolve infection relatedness at high spatial resolutions in the context of malaria elimination, particularly in settings with low prevalence and high levels of monoclonality (20, 35, 50, 51). Additionally, by combining *P. falciparum* drug resistance markers with flanking microsatellite loci, one may examine the genetic diversity and evolution of selective signatures surrounding drug resistance genes (35, 52).

This approach surpassed genetic typing approaches based on surface antigens and revealed numerous essential information about the spread of epidemics, the extent of recombination, and the degree of population differentiation (49). The use of multilocus approaches surpasses approaches using surface antigens because it is extremely widespread in *P. falciparum*, occurring every 2–3 kb throughout the genome. Thus, microsatellites can be used to better distinguish between COI in high and low transmission settings (49) and between local and imported cases. Microsatellites provide a metric that better identifies and quantifies the rate of importation and risk of local spread of malaria infections by comparing the genetic relatedness (53). The neutral evolving nature of the markers, ease of multiplexing with 10 or more markers in one PCR run, and abundance throughout the genome are some other advantages (49, 54–56). For example, this approach was able to determine the genetic diversity in a low transmission setting of the Kingdom of Eswatini (57) and differentiate between the COI of local and imported cases in Southern Africa (58).

As with *msp/glurp* typing, microsatellite typing is dependent on reliable DNA fragment amplification by PCR and can introduce artifacts in amplification and diversity estimates, thus making population structure studies painstaking. Furthermore, no method exists for resolving parasite haplotypes in mixed infections and standardization across laboratories is complicated because of the variety of methods used in genotyping microsatellites. In addition, the scarcity of markers, the high mutation rate, and the difficulty of appropriately scoring alleles (fragment sizing) reduce the resolution of microsatellite markers used to identify related parasites (54, 59).

The challenges in defining fragment sizes can be addressed using either capillary electrophoresis or next-generation sequencing (NGS) albeit at extra cost. Unlike fragment size estimation in gel electrophoresis, which is subjective, capillary electrophoresis or NGS method is based on fluorescently labeled oligonucleotides or nucleotides, separated in fine capillaries, and detected by laser. They provide more separation efficiency and higher resolution (60). Due to their enormous variability, parasite population substructure may be undetected in sites with high transmission, as panels of ten to fourteen markers may yield a false estimation of relatedness (54). The generation of *P. falciparum* genome sequences is becoming more popular as the cost decreases, allowing for the design of more effective SNP-based panels tailored to study questions and settings. However, these genomes are now available for mining novel microsatellite loci to enrich existing loci to address specific research questions such as the origin and spread of drug resistance, making it relevant for low resource settings like sSA in the long-term (26, 35).

### Single nucleotide polymorphism genotyping

Single nucleotide polymorphisms (SNPs) are regarded as one of the most common forms of genetic variation and for this reason a powerful tool for the identification of disease-causing organisms, their genetic diversity, phylogenetic analysis and outbreak surveillance. The SNP barcode generates a unique haplotype signature for each infection, distinguishing single-clone infections from those with parasite genome mixtures (35, 61). Some studies have used this technique to develop genotyping assays which incorporate SNP barcoding to evaluate parasite genotypes derived from communities, malaria patients, or laboratory strains (35, 62). SNP barcoding is also sensitive for detecting and genotyping sub-microscopic parasitaemia in locations with low malaria transmission, even in the absence of positive Rapid Diagnostic Tests (RDT) (35, 63). Additionally, the approach has the potential to identify epidemic sources (35, 64).

SNP genotyping approaches can be used on qPCR, High resolution Melting genotyping and the Sequenom Mass Array assays. These approaches have an advantage over microsatellites in that SNP data are inexpensive, easy to obtain from commonly used dried blood spot (DBS) samples, and suitable for field samples across a range of transmission settings. Thus, SNP data remain the most frequently used approach for genotyping studies (38, 65). A study on Kenyan and Gambian parasite isolates that used at least 80 SNP genotyping on the Sequenom MassARRAY iPLEX platform revealed no clear spatially confined parasite subpopulations, but rather a diffused spatio-temporal pattern to parasite genotypes (66). Another SNP approach is the TaqMan array card (TAC), a 384-well microfluidic real-time PCR system that compartmentalizes each sample into 48 separate PCRs simultaneously and has been used for the detection of multiple tuberculosis (TB) drug resistance markers, syndromic pathogen detection and has yielded 100% accuracy in detecting known antimalarial drug resistance markers compared to sequencing (67).

Some disadvantages of SNP genotyping is that it limits analyses to what is known, requires the design of primers, data is primarily interpreted per SNP and few laboratories in sSA have been able to utilize these assays due to the absence and cost of advanced PCR or array technologies (64, 68). Moreover, PCR genotyping is ineffective in detecting structural mutations that are relevant for downstream applications (26) thus, there is a need for NGS platforms to provide higher resolution and coverage of genomes.

### Targeted amplicon deep sequencing

Targeted amplicon deep sequencing (TADS) is the sequencing of specific regions of the genome using next-generation technologies. This approach increases the sample load, the processing speed and at the same time, lowers the costs of molecular analysis (69). TADS of short amplicons have the potential to overcome some of the limitations associated with long polymorphic genotyping markers, most notably the effect of a marker's fragment length on the detectability of minority clones. In transmission studies, TADS is used to differentiate indigenous infections from imported infections, especially in areas marked for pre-elimination.

To gain a better understanding of malaria epidemiology and infection dynamics, individual parasite clones are tracked throughout time to determine their acquisition, elimination, or persistence in a human host. Tracking of genotypes over time can be done by taking repeated blood samples at specific time intervals and through longitudinal cohort studies and randomized-controlled trials. TADS-based genotyping of longitudinal samples from Papua New Guinea allowed for the tracking of clone density over time. This was to investigate clone competition or the dynamics of resistance-associated genotypes, and provide an additional parameter for investigating malaria infection dynamics (49). TADS-based genotyping can also be used to determine the incidence of new clones per host and serves as a surrogate measure for the exposure of an individual and the transmission intensity in a population (24). This is important in antimalarial clinical trials, where existing clones must be identified separately from new clones in post-treatment samples from patients with recurrent parasitaemia (70, 71). Previously published studies employed two distinct techniques for genotyping *P. falciparum* and *P. vivax* using TADS:

Sequencing of conventional length polymorphic genotyping markers, such as *P. falciparum msp1* and *msp2* (70, 72). Alternatively, non-repetitive areas containing a high number of single nucleotide polymorphisms (SNPs) are sequenced, such as the *P. falciparum* circumsporozoite protein (*csp*) or the *P. vivax msp1*. The advantage of this approach is that a single sequence read connects all SNPs inside an amplicon, allowing for immediate haplotype identification.

Multiple loci of genome-wide distribution were sequenced, with each locus containing a single SNP (70, 73). This strategy is well suited for population genetic studies, as these loci are unrelated. The disadvantage is that each infecting clone's haplotype must be reconstructed, which is difficult or impossible in data with a high number of co-infecting clones per host (38, 70). Other challenges with TADS include methodological accuracy issues during the experimental procedure, guaranteeing equimolar concentrations of amplicons to minimize over-representation of certain clones per sample, and limitations of detection for minority clones based on known DNA controls.

#### Molecular inversion probes

Targeted deep sequencing is highly sensitive and offers the opportunity to multiplex in circumstances whereby epidemiological studies involve hundreds of samples, thus decreasing the cost. One such multiplexing approach is the use of Molecular Inversion Probes (MIPs), initially referred to as “padlock probes” (74). This is an enrichment process that allows for multiplexing at different stages. Interest in MIPs has increased because; 1) It is cost-effective, 2) It has high multiplexing potential, 3) It is less time-consuming as only a small number of processing steps are required to achieve targeted region capture, 4) It has low DNA requirements (50–250 ng) as compared to other target enrichment methods, 5) It has high accuracy and specificity, 6) It allows enrichment of target regions at a scale that is matched by NGS platforms (75). MIP-TADS demonstrated that *pfhrp*2/3-deleted *P. falciparum* is a common cause of false-negative Rapid Diagnostic Test (RDT) results in two locations in Ethiopia. This genomic method revealed evidence of a recent, significant selection for *pfhrp*2 deletion in the sites studied, which is cause for concern (76). The downside of this approach is, like other fragment genotyping approaches, genotyping of samples containing multi-clone infections remains an unresolved challenge when multiple genome-wide loci are targeted.

### Whole genome sequencing

Whole genome sequencing is the process of determining the entire DNA sequences in the genome of an organism. It is a highly sensitive tool that can be used to gather information about potential drug resistance, identify homologous relapses with improved accuracy, and analyze population structure and gene flow, especially as the cost of this technology declines rapidly. The advent of NGS is making WGS a standard today in the field of life sciences, as PCR genotyping and targeted sequencing provide less information compared to the whole genome (14).

NGS involves large-scale DNA sequencing without the use of gels and reversible terminators and was developed with an emphasis on speed and efficiency in response to advancing technology. Millions of reads can be generated in hours using platforms such as Illumina/Solexa, ABI/SOLiD, 454/Roche, and Helicos, which are at the fore-front of delivering unmatched opportunities for high-throughput genomic research (77). Thus, adapting WGS approaches is pertinent to study the malaria parasite and the epidemiology of the disease, as different regions are at different phases in their malaria elimination agenda (26).

Whole Genome Sequencing has provided deeper insight into the movement, demographics, and evolution of resistant parasites (78) and the opportunity to develop new control methods, including new drugs and vaccines, improved diagnostics and effective vector control techniques (79). A genome-wide analysis of polymorphism in *P. falciparum* from 24 malaria-endemic settings in 15 African countries revealed major clustering into western, central, and eastern ancestries, in addition to a highly divergent Ethiopian population (80). Whole Genome Sequencing has been used to investigate parasite diversity, with interpretations based on genome-wide diversity across various loci (24). Miotto and colleagues in 2015, through a large multicenter genome-wide association study of *P. falciparum* resistance to artemisinin, across 15 locations in Southeast Asia identified at least 20 mutations in kelch13 associated with a slow parasite clearance rate after treatment with artemisinin derivatives. This data also revealed that the *fd* (ferredoxin), *arps10* (apicoplast ribosomal protein S10), *mdr2* (multidrug resistance protein 2) and *crt* (chloroquine resistance transporter) polymorphisms are markers of a genetic background on which kelch13 mutations are especially prone to emerge, and that they correlate with the current geographical boundaries and population frequencies of artemisinin resistance (81).

Unlike individual locus analyses, which may be susceptible to strong natural selection, inferences from genome-wide diversity are less subject to target bias and so more correctly reflect broad patterns of *P. falciparum* genetic variation (35). However, the cost is a significant impediment to implementing WGS and it remains impractical in many situations due to technical constraints connected with large-scale data storage and processing. These studies cannot usually report diversity statistics due to multiple infections in their samples (82).

## Recommendations for application of genomics techniques in resource-limited contexts

The incorporation of genomic data into malaria surveillance is a recent development, facilitated considerably by the continuous rise in accessibility to genomic technologies. However, sSA continues to lag behind other regions in the translation of findings to direct control and elimination efforts for a number of reasons. Given that one of the recommendations of the Malaria Eradication Research Agenda (malERA) expert panel is to improve the characterization of the parasite reservoir and develop new tools to assess transmission (10), some of the aforementioned tools should be made available for routine surveillance where useful. To close the gap between sSA and other regions and fully utilize the potential of genomic tools for measuring transmission, several challenges will have to be resolved. These include but not limited to funding, slow reagent supply chain, insufficient local infrastructure, challenges with integrating into routine surveillance systems, lack of technical expertise and staff retention after Bioinformatics training (69).

The restricted infrastructure is the first problem that needs to be solved. Lack of assistance provided by local governments in sSA and, to a lesser extent, donor fatigue internationally are major contributors to this problem. As we work toward malaria elimination and eradication, governments, researchers, NMCPs, and other important stakeholders must work together to use genomics to track malaria parasites. The NMCPs should be heavily involved in the development and execution of genomic investigations to make sure that they address issues of relevance and priority in the local environment. But before that can happen, researchers need to educate all stakeholders on the added value of genomic data and how it may be integrated into routine program activities including surveillance. The COVID-19 experience exposed everyone to some fundamental genomics, which may be leveraged to improve stakeholder participation in malaria genomics as a whole and transmission monitoring specifically. As a result, the time is right to achieve this. This will guarantee that the NMCPs include genomics in their strategic plan, and genomes data will be widely used to support malaria elimination initiatives. Governments will also be eager to assist this worthwhile course using the COVID-19 experience as a model. To close the human resource capacity gap, more genomics and bioinformatics training should be promoted throughout Africa. Technical experts should be kept on staff and paid fairly. They can also be engaged remotely to analyze data. Bulk purchasing and negotiating directly with manufacturers as a sub-region can improve the procurement of reagents and supplies, as demonstrated by the Pathogen Genomics Initiative (PGI) of Africa CDC during COVID-19.

Depending on financing support and the stage of a country (malaria elimination or control), a genomic strategy to monitor malaria transmission must be chosen. A reasonably inexpensive, reliable, simple but low through-put method for analyzing the SNPs to monitor malaria transmission at the control stage is the Taqman based real-time PCR assay and high-resolution melting genotyping (83, 84). In reference labs in malaria-endemic areas, the technique can be simply applied with the right staff and facilities.

In general, NGS techniques (WGS, TADS) will provide additional information, particularly when used to analyze the population dynamics and structure of parasites at a lower scale (15, 85). Although whole genome sequencing provides comprehensive information on the genetics of the parasite, it is better suitable for pre-elimination situations. During the pre-elimination era, WGS will give insights into the local and/or regional parasite populations to guide the selection of the right markers to be used in the elimination phase. On the other hand, TADS would be more economical and would offer the most pertinent data at both the control and elimination settings. Nonetheless, to analyze sequencing data, especially NGS, complex infrastructure with sufficient computer capacity and highly skilled employees are needed.

The creation of national and sub-regional reference labs could reduce the amount of equipment and qualified personnel needed. Malaria genomics for monitoring transmission dynamics in sSA should learn from the very effective PGI COVID-19 approach for a successful implementation. It will take coordinated efforts from various stakeholders at the national, sub-regional, and international levels to create the necessary framework for the establishment and maintenance of these reference laboratories, as well as cost-effective planning to ensure the efficient use of available resources. The Pathogen Diversity Network Africa (PDNA) collaboration model and the Malaria Genomic Epidemiology (MalariaGEN) consortium sample collecting, storage, or data exchange approaches will be helpful in this situation (86, 87).

In conclusion, we believe that the time is right to incorporate genomics into surveillance of malaria transmission in sSA. The successful application of malaria genomics for the monitoring of transmission across Africa will be ensured by effective planning and implementation. This includes the development and sharing of standardized operational procedures (SOP), consolidated procurements across sSA, training a cadre of bioinformaticians and genomics experts, and a strong stakeholder engagement. Using lessons learned from the COVID-19 sequencing projects across Africa, we can start with TAD and move on to WGS as costs continue to further lower and WGS can be operationalized into routine program activities.

## Author contributions

AG suggested the topic and contributed to the writing and review of the manuscript. BM mainly wrote the first draft of the manuscript and worked on the review. NA-B contributed to the writing of the first draft and was involved in the review process. All authors contributed to the article and approved the submitted version.

## Funding

AG was funded by the Bill and Melinda Gates Foundation as a Calestous Juma Science Leadership Fellow. Investment ID INV-037564.

## Conflict of interest

The authors declare that the research was conducted in the absence of any commercial or financial relationships that could be construed as a potential conflict of interest.

## Publisher's note

All claims expressed in this article are solely those of the authors and do not necessarily represent those of their affiliated organizations, or those of the publisher, the editors and the reviewers. Any product that may be evaluated in this article, or claim that may be made by its manufacturer, is not guaranteed or endorsed by the publisher.
